# Imaging Methods in the Diagnosis of Optic Disc Drusen

**DOI:** 10.4274/tjo.66564

**Published:** 2016-10-17

**Authors:** Betül Tuğcu, Hakan Özdemir

**Affiliations:** 1 Bezmialem Vakıf University Faculty of Medicine, Department of Ophthalmology, İstanbul, Turkey

**Keywords:** Optic disc drusen, imaging methods, optical coherence tomography

## Abstract

Optic disc drusen (ODD) are benign congenital anomalies of the optic nerve characterized by calcified hyaline bodies. While superficial drusen can be diagnosed easily during fundus examination, detecting buried drusen requires the use of additional imaging methods such as B-scan ultrasonography (USG), fundus fluorescein angiography (FFA), computed tomography (CT) and fundus autofluorescence (FAF). ODD can be detected by USG with the presentation of highly reflective round structures. ODD appear as hyperautofluorescent areas on FAF and bright spots on CT scans. FFA can be helpful in differentiating ODD from true optic disc edema. Optic disc edema shows early hyperfluorescence due to diffuse leakage whereas ODD presents as well-defined hyperfluorescence in the late phase. In recent years, it has been reported that optical coherence tomography (OCT) examination has allowed more detailed evaluation of ODD and yielded useful findings for the differentiation of optic disc edema from ODD. In this review, the role of OCT in the diagnosis of ODD is discussed.

## INTRODUCTION

Optic disc drusen (ODD) are autofluorescent, calcified deposits found in the optic nerve head, and typically occur in small, crowded optic discs.^1^ Their prevalence ranges from 3.4 to 24 per 1,000 according to clinical studies and 1-2.4% in histological studies.^2,3,4^ The prevalence of ODD is higher in women and involvement is usually bilateral.^5,6^

Although the mechanism of drusen formation has not been fully determined, it is believed that congenitally small disc and scleral channels may cause axoplasmic flow stasis and ganglion cell axon death.^7^ Furthermore, it has been proposed that drusen continue to grow and move toward the disc surface due to ongoing neural tissue loss. This is supported by the fact that visual field defects, which progress with age, are often detected in the second decade of life.^8^ Ophthalmic and systemic diseases commonly associated with drusen are retinitis pigmentosa, angioid streaks, Usher syndrome, Noonan syndrome and Alagille syndrome.^2^

ODD is usually overlooked in clinical examinations because it does not cause any visual symptoms; visual functions are generally not affected early in life. Visual field anomalies arising due to ODD are often not noticed by patients. Visual field defects are reported to occur less often with buried ODD compared to those which are more superficial.^9^ ODD-associated visual field defects arise in the inferonasal quadrant in particular, and may manifest as blind spot enlargement, concentric constriction, arcuate defects or peripheral vision loss.^10^

Rarely, ODD can lead to vision loss, usually in the form of a slight decline in visual acuity.^11^ The most common cause of sudden vision loss associated with ODD is nonarteritic anterior ischemic optic neuropathy (NAION). Compared to the typical NAION patients, ODD patients are younger and have better visual prognosis.^12^ Other rare vascular complications arising due to ODD that have been reported in the literature include subretinal neovascularization, central retinal artery and vein occlusion.^13,14,15^ ODD may lead to hemorrhage in the retina and disc margin. Optic disc hemorrhages in particular are more common in children.^16,17,18^

**Figure 1:**
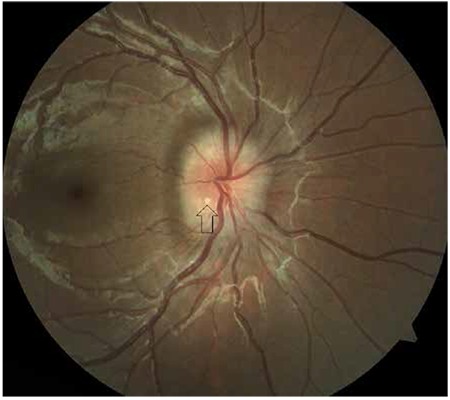
Superficial drusen located in the inferior optic disc appear as smooth-edged, round deposits (arrows)

**Figure 2:**
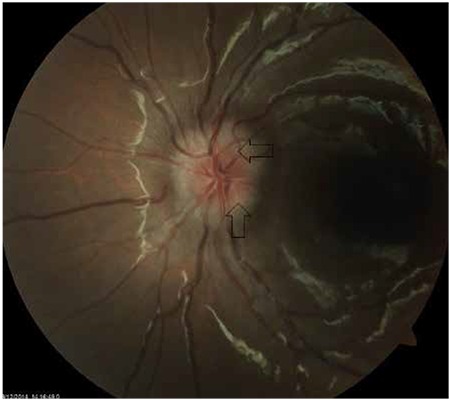
Appearance of buried drusen (arrows) located near the lamina cribrosa on ophthalmoscopic examination

**Figure 3:**
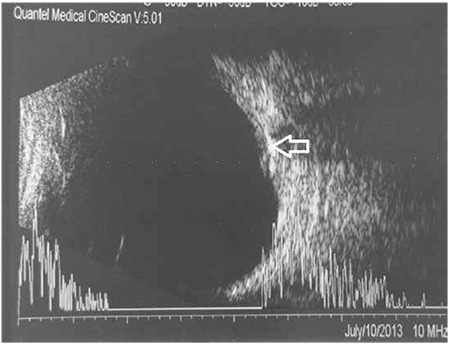
Buried drusen (arrows) have hyperechogenic appearance on B-scan ultrasonography

**Figure 4:**
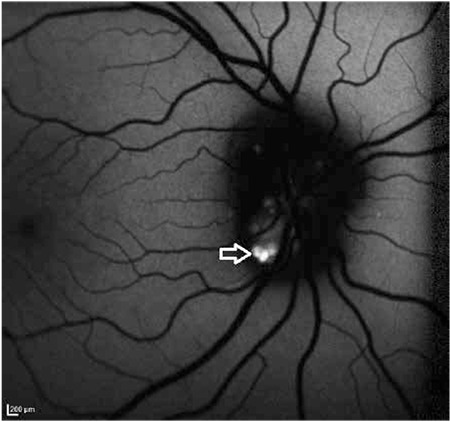
Superficial, round or oval drusen appear as hyperautofluorescent structures with irregular borders on fundus autofluorescence examination

**Figure 5:**
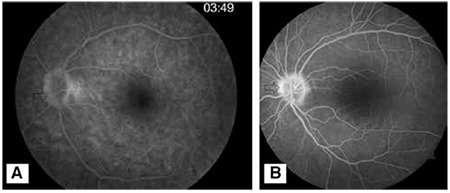
On fundus fluorescein angiography, drusen appear as smooth-bordered hyperfluorescence at late phase (A), whereas optic disc edema shows early diffuse leakage with irregular margins (B)

In clinical practice, it can be extremely difficult in some cases to differentiate ODD from true optic disc edema, which is a critical distinction in terms of treatment approach. Superficial ODD can be easily identified as round deposits in ophthalmoscopic examination (Figure 1), whereas those located closer to the lamina cribrosa (Figure 2) may not be evident in this examination, requiring additional imaging modalities to confirm diagnosis.^19^ One of these is B-scan ultrasonography (USG), which is an inexpensive, fast and practical method of reliably and effectively diagnosing ODD. ODD are easily diagnosed by B-scan USG due to their inherent high reflectivity (Figure 3). The major advantage of USG is the ability to show even the posterior borders of buried drusen, but its drawbacks are low resolution and inability to provide data on the neural retina.^20,21^ Although fundus autofluorescence (FAF) is a convenient method of visualizing more superficial drusen, it is insufficient for detecting buried drusen. Superficial drusen appear on FAF as round or oval hyperautofluorescent structures with irregular edges (Figure 4). Drusen at different levels show different intensity of hyperautofluorescence. Deeply buried drusen do not appear on FAF because the overlying tissue prevents autofluorescence. Fundus fluorescein angiography (FFA) is a more difficult and invasive procedure than FAF, but can be utilized in selected cases when differentiation of deeper ODD from optic disc edema is challenging. On FFA, eyes with ODD exhibit mild hyperfluorescence with smooth margins in the peripapillary area in the early phase which becomes more pronounced in the late phase. In optic disc edema and papilledema, hyperfluorescence is evident in the early phase due to diffuse leakage (Figure 5a and 5b). The most distinctive difference between ODD and papilledema is the absence of telangiectatic vessels at the optic nerve head in ODD. Furthermore, unlike in ODD, leakage appears in the early phase as spots on the disc surface which later coalesce.^2^

**Figure 6:**
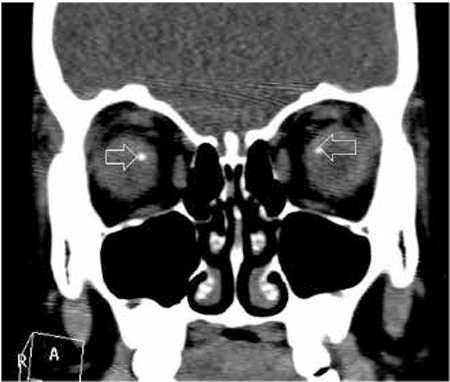
Bilateral optic disc drusen (arrows) appear white and shiny with smooth borders

Computed tomography (CT) can also be utilized to detect buried drusen. Drusen appear as bright white bodies on CT due to their calcium content (Figure 6). In routine clinical CT examinations, scans are done in 1.5 mm sections, but a thorough scan using thinner sections should be performed in order not to miss small drusen. Because CT is not as sensitive as USG and involves radiation exposure, it is only used in the rare instances that other imaging modalities are not adequate.^2^ Furthermore, buried drusen which are not calcified may not appear on fundoscopy, USG or CT.^22^

ODD are usually located on the nasal side of the optic disc. In some cases, they can lead to extensive, severe optic disc swelling, simulating optic nerve tumors.^23^ It can be extremely difficult to differentiate ODD from the shiny particles seen in chronic papilledema.^24^ The coincidence of ODD and glaucoma may make evaluation of the optic disc and visual field challenging. Although drusen may not block the development of glaucomatous cupping in such cases, the presence of a small, crowded disc may mask glaucomatous cupping.^25^ Furthermore, it may not be possible with visual field testing to determine whether nerve fiber damage is a result of glaucoma or ODD. For this reason, objective evaluation of the nerve fiber layer by optical coherence tomography (OCT) is necessary, especially in patients without glaucomatous cupping.^26^

OCT allows the early detection of retinal nerve fiber layer (RNFL) thinning. Its advantages include the ability to quantitatively assess nerve fiber loss and its high degree of repeatability.^27^ OCT provides more objective data in the evaluation of RNFL loss due to ODD compared to the subjective method of red-free photography.^28^ OCT studies on this topic have demonstrated that RNFL thinning is most pronounced in the nasal peripapillary region, where ODD are most commonly found. RNFL values are often normal in cases of buried ODD, but RNFL thinning has been observed in all peripapillary quadrants in cases with superficial drusen.^2,29^ In contrast, Gili et al.^30^ did not find significant thinning in the temporal quadrant; they attributed this to the less common occurrence of drusen in the temporal disc. In the same study, they also observed a significant association between RNFL thinning and visual field defects. In a very recent report, macular ganglion cell layer (GCL) thickness and RNFL both decreased significantly with superficial drusen, while GCL thickness decreased more than RNFL thickness in buried drusen. The authors emphasized that GCL analysis was more sensitive than RNFL in the detection of axon damage seen with drusen.^31^

**Figure 7:**
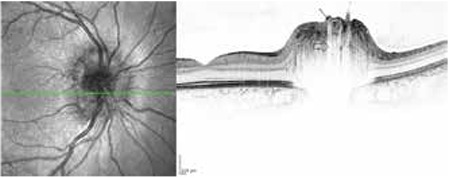
Appearance of superficial optic disk drusen on spectral-domain-optical coherence tomography (arrow indicates superficial drusen; bold arrow indicates hyporeflective shadow)

Because of the low resolution of time-domain OCT, detailed analysis of ODD have only been possible in clinical studies using high-resolution spectral-domain OCT (SD-OCT).^32,33^ Substantially different results were reported in these studies regarding the shape, size, and reflective properties of ODD, which was proposed to be a result of variations in the anatomic position and composition of drusen.^33,34,35^ Superficial drusen are reported to appear hyporeflective and have a shadow effect, whereas buried drusen appear hyperreflective on SD-OCT (Figure 7).^36^ Despite the high resolution of SD-OCT, it may still be inadequate for the visualization of deeper ODD.^37^ It is difficult to detect the posterior border of drusen on OCT because the resolution decreases as depth increases and the hyperreflective anterior surface of ODD causes a shadowing effect.^19^ The biggest problem in evaluating optic disc lesions with OCT is the presence of nerve fibers and dense vasculature at the disc surface causing hyperreflectivity and shadowing. In some cases, it is not easy to distinguish calcified drusen and their shadows from large superficial blood vessels on OCT.^22^

Recent literature has provided new SD-OCT findings which may assist clinicians in differentiating optic disc edema from buried ODD in particular.^36,37,38,39,40,41,42^ Optic nerve head elevation can be seen on OCT in both clinical conditions, but in disc edema the inner surface of the optic nerve has a smooth edge, whereas in ODD the surface is bumpy and has been termed ‘lumpy-bumpy’ in the literature. The triangular subretinal hyporeflective space (SHS) between the sensory retina and retinal pigment epithelium (RPE) has been reported to have a larger area and thickness in disc edema compared to ODD.^39,40,41^ Furthermore, Johnson et al.^41^ pointed out that the SHS gradually thins moving away from the optic disc in papilledema patients, whereas its thickness decreases suddenly and dramatically in patients with ODD. Kupersmith et al.^43^ also demonstrated that in papilledema, the RPE and Bruch’s membrane are deformed inward toward the vitreal space due to elevated pressure in the retrolaminar subarachnoid space. Another study determined that total retinal thickness, between the internal limiting membrane and the RPE, is a more sensitive and important parameter than RNFL in the differentiation of papilledema and ODD. In papilledema, a greater increase in total retinal thickness was observed compared to RNFL due to peripapillary subretinal fluid.^44^

**Figure 8:**
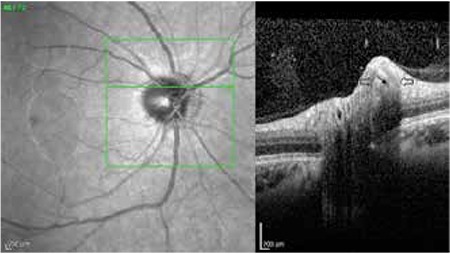
Enhanced depth imaging-optical coherence tomography image of drusen in the superonasal optic disc showing internal hyperreflective focus, external hyperreflective border and hyporeflective area in between (open arrows indicate the drusen borders; arrow shows hyporeflective area)

New OCT technologies developed in recent years [enhanced depth imaging, (EDI)-OCT and swept source, (SS)-OCT] have improved our ability to examine the form and structure of drusen anatomically. EDI-OCT and SS-OCT allow the detailed examination of the area between the RPE and lamina cribrosa, which could not previously be visualized using SD-OCT. With its high-resolution capability, this new technology enables a closer evaluation of the internal structure of drusen and their relationship with the lamina cribrosa.^19^ EDI-OCT allows the examination of structures 500-800 µm deeper than possible with conventional OCT. Furthermore, because the posterior margins of ODD can be better determined by EDI-OCT, their area and volume can also be calculated.^21^ It has been reported that drusen have a central hyperreflective focus and an outer hyperreflective edge, with a hyporeflective area in between (Figure 8). A negative correlation between drusen diameter and RNFL thickness as well as greater RNFL loss in drusen located in the optic canal have been demonstrated. In addition, greater RNFL thinning was observed in the presence of drusen with internal hyperreflective foci. In recent years, it has been reported that EDI-OCT and SS-OCT are superior to USG in the detection of buried drusen.^38^

SS-OCT technology has enabled more detailed evaluation of ODD compared to standard SD-OCT.^45^ The findings regarding ODD are similar to those of EDI-OCT studies. However, the SS-OCT studies are few and do not include enough patients. Although better penetration and resolution can be achieved using different wavelength lasers, this OCT technology has not yet become widely used in clinical practice.^19^

Even if followed without treatment, ODD patients should still be closely followed over the long term for possible complications. Detailed examination of ODD structure and location enabled by recent developments in OCT technology have bettered our understanding of the relationship between ODD, RNFL loss and visual field defects. Because the differential diagnosis of ODD includes papilledema and optic neuropathies causing disc edema, correctly diagnosing these patients is crucial to avoid unnecessary treatment and surgery. Although superficial drusen can sometimes be readily identified in a careful fundus examination, cases with buried drusen may require all of the methods described above to reach a definitive diagnosis. OCT technology has substantially facilitated differential diagnosis in these cases and with continuing improvements will undoubtedly have an even more important place in ODD diagnosis and follow-up in the future.

### Ethics

Peer-review: Externally peer-reviewed.
